# An mHealth Assistive System “MyLung” to Empower Patients with Chronic Obstructive Pulmonary Disease: Design Science Research

**DOI:** 10.2196/12489

**Published:** 2019-03-19

**Authors:** Riad Alharbey, Samir Chatterjee

**Affiliations:** 1 Information Systems and Technology College of Computer Science and Engineering University of Jeddah Jeddah Saudi Arabia; 2 Center for Information Systems & Technology Claremont Graduate University Claremont, CA United States

**Keywords:** assistive technology, patient empowerment, chronic obstructive pulmonary disease, design science research

## Abstract

**Background:**

Chronic obstructive pulmonary disease (COPD) comprises a group of progressive diseases that deteriorate lung functions. When patients cannot breathe, nothing else in their lives matter. Breathlessness has negative implications on patients’ lives, which leads to physical and psychological limitations. Moreover, the lack of relevant and updated information about the causes and consequences of the disease can exacerbate the problems of health literacy, information accessibility, and medical adherence.

**Objective:**

The objective of this study is to design an innovative mobile health (mHealth) app system called “MyLung” that provides complete solutions in order to increase self-awareness and promote better self-care management. This system, an information technology artifact, includes three novel integrative modules: education, risk reduction, and monitoring.

**Methods:**

The utility and effectiveness of the assistive mobile-based technology were evaluated using a mixed-methods approach. The study combined quantitative and qualitative research methods to thoroughly understand how the assistive mobile-based technology can influence patients’ behavioral intention to change their lifestyle. Thirty patients were categorized into two groups (intervention group and control group).

**Results:**

The results from the quantitative analysis led to four follow-up interviews in the qualitative study. The results of the quantitative study provided significant evidence to show that the design of MyLung leads to a change in the awareness level, self-efficacy, and behavioral intention for patients with COPD. The *t* tests revealed a significant difference before and after using the mobile-based app with regard to the awareness level (mean 3.28 vs 4.56; *t*_10_=6.062; *P*<.001), self-efficacy (mean 3.11 vs 5.56; *t*_10_=2.96; *P*=.01), and behavioral intention (mean 2.91 vs 4.55; *t*_10_=3.212; *P*=.009). Independent sample *t* tests revealed significant differences between the intervention group and the control group in terms of the awareness level (mean 4.56 vs 3.31; *t*_19_=4.80; *P*<.001) and self-efficacy (mean 5.56 vs 3.66; *t*_19_=2.8; *P*<.01). Integration of findings from quantitative and qualitative studies reveled the impact of the design in a comprehensive manner. These inferences are referred to as “meta-inferences” in this study.

**Conclusions:**

We designed an innovative assistive mobile-based technology to empower patients with COPD, which helped increase awareness and engage patients in self-care management activities. The assistive technology aims to inform patients about the risk factors of COPD and to improve access to relevant information. Meta-inferences that emerge from the research outputs contribute to research into chronic management information systems by helping us gain a more complete understanding of the potential impacts of this proposed mobile-based design on patients with chronic disease.

## Introduction

### Background

Chronic obstructive pulmonary disease (COPD) is a prevalent disease worldwide and a major cause of morbidity and mortality [1]. COPD is a group of obstructive lung diseases encompassing multiple conditions that share two common factors: airflow limitation and unusual inflammations in the lung. Patients with COPD experience symptoms such as chronic cough, sputum production dyspnea, chronic sputum, hyperinflation, and wheezing [1]. Several factors are responsible for triggering COPD symptoms and are based on two categories: lifestyle (typically, long-term smoking) and occupational or environmental factors. Patients with COPD are susceptible to the risk of frequent episodes or exacerbations. According to Celli et al [2], exacerbation is defined as “sustained worsening of the patient’s condition from the stable state and beyond normal day-to-day variations that is acute in onset.”

Because COPD conditions have a long-term effect, patients frequently suffer from complications and require constant education and awareness to promote better empowerment [3]. Education empowerment increases patients’ knowledge of matters associated with their conditions [4]. Patients with chronic diseases should receive an effective education on how to actively manage their condition on a daily basis. Equipping patients with education empowerment mechanisms about their chronic conditions, such as understanding the consequence of risks and receiving timely guidelines, has been associated with improved self-management and self-control of disease [5]. These mechanisms will help patients change their behaviors with regard to managing their disease conditions [5].

Access to health care information and educational resources becomes a challenge for patients with COPD because of the barriers that prevent easy delivery of information. These barriers result from physiological factors relevant to patients, environmental settings, and cost of educational programs [6]. Physiological barriers play a major role in the way a patient with COPD is able to perceive and process health information [7]. The consequences of COPD can be better managed with information technology, in particular, mobile health (mHealth) and telemedicine. mHealth technologies can enable a variety of technical features that facilitate better education and self-management for patients with COPD, which, in turn, persuade patients to change their behavioral intentions toward self-care. Features included in such mobile technologies can be utilized for health-related purposes. They should be useable by all types of patients, including the elderly, people with low literacy, and people with disability [8]. To give an illustrative example, a patient can use a mobile app to plan daily activities in advance and will receive notification messages or short videos to remind him/her of the activities. Additionally, patients can monitor their symptoms and vitals when related data are collected from the sensors through the use of Bluetooth.

COPD can lead to behavioral problems [5] that increase the threat of disease progression. These problems are related to daily lifestyle and medication-taking behaviors. Behavioral problems occur due to a lack of access to appropriate skills and knowledge that can help patients better manage their conditions [9]. The consequences of breathlessness are manifested as functional impairment that leads to psychological limitations. Patients with COPD always experience anxiety, which causes depression and distress [10]. When patients panic about the fear of becoming breathless, they cannot be easily motivated to engage in self-care behavioral activities. Additionally, better lifestyle behaviors can be accomplished when patients receive recommendations about outdoor pollution hazards, smoking cessation techniques, and regular exercises. Relevant knowledge and skills help develop self-efficacy, which increases patients’ confidence in their abilities to perform behavioral actions.

A behavioral change–support system (BCSS) has become an attractive tool for information system researchers to explore and understand the mechanism of empowerment education for adult learners. BCSS has been found to be an important agent of change at individual and social levels [11]. When the learning and recommendation content of interventions is designed with respect to BCSS strategies, the intervention will influence patients’ engagement and behavioral change through a real-life experience. With the use of such strategies, the self-management intervention tool for COPD can include features such as task compression and follow-up looping [11].

The objective of this research is to design and develop an innovative mobile-based technology that offers a complete solution for patients with COPD. This design would resolve the issues of lack of awareness about the risk factors of COPD, information accessibility, and management of COPD symptoms among patients with COPD. The problems are investigated through the lens of design science research [12,13], which is a research approach that aims to design and evaluate an innovative technology [12] and involves processes that consult scientific theories (ie, health belief model) [13]. Design science research sheds light on patients’ needs and creates an effective assistive technology to fulfill those needs. The innovative design introduces three modules with complete and integrated features. The design offers education, risk reduction, and monitoring modules in one artifact called “MyLung.” Therefore, the questions of this study drive the design process to create an effective information technology artifact. We aimed to address the following main research question: How can an effective integrative assistive mobile-based technology be designed to improve patients’ understanding of COPD (knowledge about the risk factors and consequences of COPD) and to increase patient behavioral intention toward self-care?

### Prior Work

Telemonitoring and mHealth technologies are relatively new fields in COPD research [14]. Tabak et al [15] reported a pilot study on the use of a telehealth program for patients in the stable stage of COPD. The telehealth program consisted of different features including Web-based exercise and self-management of COPD exacerbations. The system helped patients measure their physical activities via an accelerometer-based activity sensor. The physical activities were presented on the Web portal. In order to manage any exacerbation, patients were asked to fill in their diary on the Web portal which, in turn, fed the decision algorithm to detect the exacerbation. Although the study provided a solution to manage patient exacerbations, the design of the system lacked simplicity. Hardinge et al [16] reported the findings of a 6-month cohort study of COPD patients’ use of an mHealth app adapted to run on an Android tablet. The app was designed by a multidisciplinary team that included primary care physicians, respiratory nurses, a secondary care respiratory physician, a psychiatrist, and engineers. The app allowed patients to self-report their symptoms. Neither of the abovementioned studies showed promising results on the effectiveness of app usage. The failure to detect a significant effect on adherence to daily activities may be attributed to the process of designing a solution that lacks motivation and engagement mechanisms. Additionally, both designs lacked empowerment and behavioral motivation elements.

## Methods

### Design

**Figure 1:**
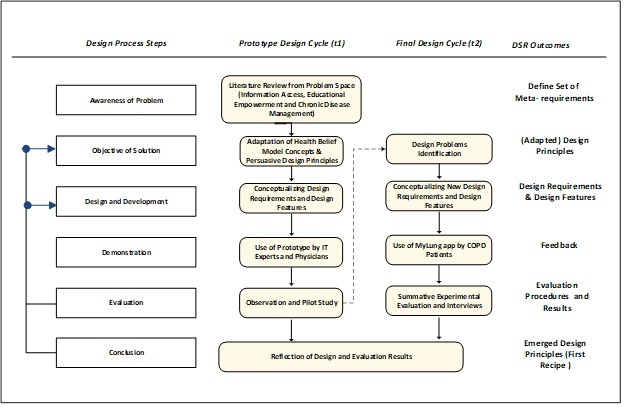
Design science research (DSR) cycles. IT: information technology.

The mobile-based technology follows design science research [12,13]. Design science research is well established in information systems research that aims to design and implement innovative technologies through design cycles [12]. The research design comprises two design cycles, as illustrated in Figure 1. The first cycle in this research project is referred to as a prototype design cycle, and the second cycle is referred to as the final design cycle.

The first design cycle was the prototype design, which started by defining a specific research problem. The research problem focuses on issues related to educational empowerment and lack of information accessibility for patients with COPD. To establish problem awareness, an initial literature review was conducted, guided by the research problem, in order to introduce a set of meta-requirements. To determine the gap in the literature, four domains were considered from the problem, namely, information access, patient educational empowerment, chronic disease management, and mobile health. Guided by the problem-awareness step, we used the health belief model (HBM) theory and BCSS principles to govern both the design requirements and process. Using knowledge gained from the literature and kernel theories, preliminary design requirements were conceptualized. Subsequently, the design requirements were translated into designed features for prototype implementation. A demonstration session was then conducted with a COPD physician, in which he examined the prototype and provided more design requirements that fit patients’ needs. To collect more feedback on the effectiveness and usability of the mobile-based prototype, we conducted a focus group interview with information technology and health experts. During the focus group session, each participant observed the prototype and offered feedback through an online survey. The new requirements and feedback initialized a new design cycle for better implementation of innovative mobile assistive technology design.

**Figure 2:**
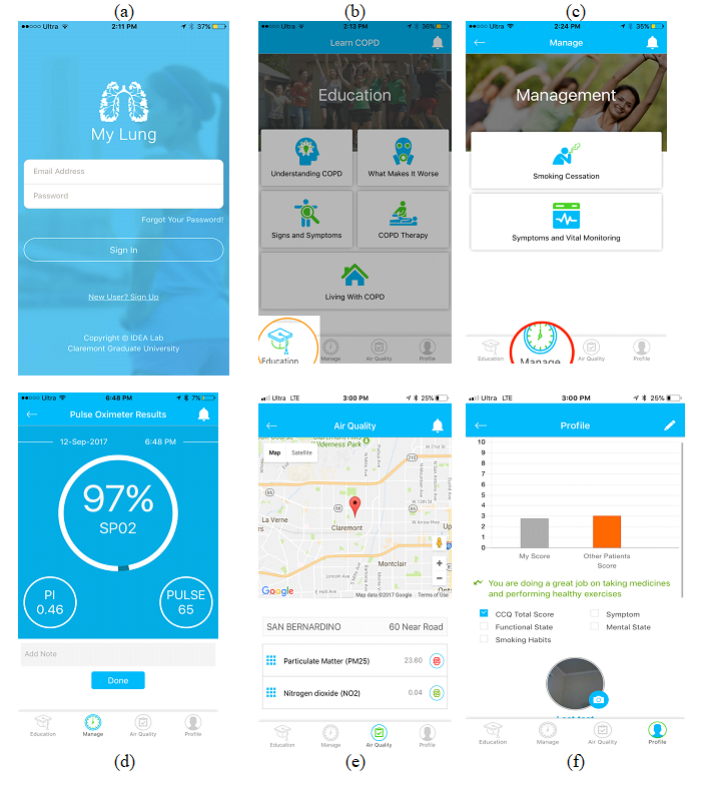
Screenshots from the MyLung mobile app.

In the second design cycle, the adapted requirements were conceptualized to guide development of the final designed features. Thus, we returned to the literature and consulted the BCSS principles to address new suggested requirements. The features were designed to provide a solution within one information technology artifact, which we called “MyLung,” and the design features were implemented to improve the quality of life for COPD patients. The information technology artifact included an educational module, a risk-reduction module, and a monitoring module. The educational module was designed to increase patient’s level of understanding about COPD by providing reliable educational videos and information. The risk-reduction module comprised features that empower patients with knowledge about ways to avoid risk-related factors. The monitoring module included features that allow patients to self-monitor their symptoms and vitals, including peripheral capillary oxygen saturation (SpO_2_). The SpO_2_ value is entered through a medical device via a Bluetooth low-energy connection, while the symptoms are entered using a clinical COPD questionnaire. The monitoring module also provides a dashboard that helps caregivers and physicians intervene before exacerbations occur. Figure 2 shows screenshots of the mobile-based app that was used by real patients with COPD.

### Procedure

The evaluation study started after obtaining institutional review board approval from Claremont Graduate University and used a mixed-method approach and a sequential explanatory design: Quantitative data were first collected from the questionnaires that were distributed through Qualtrics (Seattle, WA), following which qualitative data were collected in semi-structured interviews. A purposive sampling was used to select patients with COPD who visited a pulmonary medicine clinic in southern California. With purposive sampling, patients were selected based on the research criteria listed below. Subsequently, patients were assigned to one of two groups (intervention and control groups) without the researchers knowing about patients’ behavioral intentions. The intervention group received the MyLung app, and the control group received booklet-based information. We randomly assigned 15 patients to each group; 9 patients dropped out of the study, leaving a final sample of 21 patients (11 patients in the intervention group and 10 patients in the control group). On an average, the patients were elderly: 9% were 33-44 years old, 6% were 45-54 years old, 33% were 55-64 years old, 19% were 65-74 years old, and 33% were older than 75 years. Of the total, 52% were female. All patients spoke English at least somewhat fluently and were fairly educated, with 10% having bachelor’s degrees, 38% having associate degrees or at least some college education, 42% having a high school diploma, and 10% having less education than a high school diploma. We started the subject recruitment process and collected data in October 2017.

The inclusion criteria were as follows: receipt of a COPD diagnosis, acceptable literacy level to read and deal with a smartphone, and possession of a smartphone (Android or iPhone).

Patients in the intervention group downloaded the MyLung app from Google Play or the Apple App Store, received an iHealth Pulse Oximeter (iHealth Labs Inc, CA), and participated in a training session. During the training, we made sure that patients were able to connect the app with the pulse oximeter and understand the app features. Additionally, patients who were assigned to the intervention group were given instruction guides on how to use the MyLung features, whereas patients in the control group were given a booklet comprising traditional educational material about COPD. Prior to the training session and participation in the experiment, patients in both groups had to complete a presurvey. The survey consisted of 11 questions and was administered to patients with COPD to measure their awareness level and knowledge of COPD, self-efficacy, perceived severity, and overall intention to engage in a healthy behavioral style. The postsurvey was sent to patients 1 month after the recruitment day. Each patient in both groups received an exit survey questionnaire that was similar to the presurvey. The results from the quantitative analysis led to several follow-up interviews used in the qualitative study. The qualitative analysis yielded rich insight into phenomena related to app experience and behavioral change for patients with COPD.

### Measurements

In this section, each of the measurements is explained in detail. The measurements include the level of understanding, perceived severity, self-efficacy, and behavioral intention. The scales used for those measurements are derived from validated instruments.

#### Perceived Awareness

To measure the change in the level of awareness about COPD, an existing, validated questionnaire was adapted. The understanding COPD questionnaire (UCOPD) assesses patients’ understanding of COPD including their recognition of COPD exacerbations [17]. The instrument includes six items designed to assess patients’ perceptions of COPD. The items are rated on a Likert-type 5-point scale for each response (strongly disagree=1, disagree=2, neither agree nor disagree=3, agree=4, and strongly agree=5). The mean score for the responses of those six items was computed and used for the analysis.

#### Self-Efficacy

“Self-efficacy” is one of the HBM determinants that explains behavioral intention. The construct reflects patients’ belief in their ability to perform behavioral tasks such as physical exercises. In this study, we selected 10 measurement items from a validated and reliable COPD self-efficacy scale described by Wigal et al [18] to measure the self-efficacy level. We selected those items because they show high factor loading. In the context of COPD, the measurement of self-efficacy determines how confident patients are in managing their breathing difficulty or avoiding breathing difficulty in different situations. The items are rated with a 7-point Likert scale (from extremely uncomfortable=1 to extremely comfortable=7). The mean score for the responses of those 10 items was computed and used for the analysis.

#### Perceived Severity

“Perceived severity” reflects how patients perceive the consequences of COPD. Perceived severity is measured using a scale adapted from the HBM instrument by Champion [19]. The instrument determines a patient’s level of perceiving the seriousness of COPD. The instrument includes eight items rated on a 5-point Likert scale (from strongly disagree=1 to strongly agree=5). The mean score for the responses of those eight items was computed and used for the analysis.

#### Behavioral Intention

Behavioral intention toward self-care is reflected from the patient’s engagement in performing physical exercises and his/her intention to avoid related risk factors (eg, avoiding outdoor pollution). Behavioral intention is measured using a 1-item scale: “I intended to engage in the COPD recommended behavior.” The response to this item was rated on a 5-point Likert scale (from strongly disagree=1 to strongly agree=5).

## Results

The pre-exposure analysis showed that all patients with COPD in the two groups had similar perceptions about COPD, thus indicating that the sample was representative and there was no bias in sample selection (Table 1).

### Quantitative Results

The differences before and after use of the MyLung app/booklet are presented in Table 2. The differences between the two groups after use of the MyLung app/booklet are presented in Table 3.

The paired-sample *t* test revealed that the difference in the UCOPD score for the awareness level before and after using the MyLung app was significant in the intervention group as compared to the control group (mean 3.28 vs 4.56; *t*_10_=6.062; *P*<.001). Similarly, the independent sample *t* test revealed that the difference in the UCOPD score between the intervention group and the control group was significant after using the MyLung app (mean 4.56 vs 3.31; *t*_19_=4.80; *P*<.001). A paired-sample *t* test revealed that the difference in the self-efficacy score before and after using the MyLung app was significant in the intervention group as compared to the control group (mean 3.11 vs 5.56; *t*_10_=2.96; *P*=.014). In addition, the independent sample *t* test (Table 3) revealed that the difference in the self-efficacy score after using the MyLung app between the intervention group and the control group was significant (mean 5.56 vs 3.66; *t*_19_=2.8; *P*<.012). Although there were marginal differences in the perceived severity score between groups before and after using the app, statistical analysis showed that the difference between the mean scores was not significant. As shown in Table 2, a paired-sample *t* test revealed that the difference in the perceived severity score before and after using the MyLung app in the intervention group was not significant as compared to the control group (mean 3.03 vs 3.28; *t*_10_=0.540; *P*=.60). Similarly, the independent sample *t* test (Table 3) revealed that the difference in the perceived severity score after using the MyLung app between the intervention group and the control group was not significant (mean 3.28 vs 2.96, *t*_19_=0.864, *P*=.39). As seen in Table 2, a paired-sample *t* test revealed that the difference in the behavioral intention score before and after using the MyLung app in the intervention group was significant as compared to the control group (mean 2.91 vs 4.55; *t*_10_=3.212; *P*=.009). However, the independent samples *t* test (Table 3) showed that the difference in behavioral intention score after using the MyLung app between the intervention group and the control group was not significant (mean 4.55 vs 3.60; *t*_19_=2.05; *P*=.054). This result might be related to the small number of subjects included in the study. Additionally, Table 2 reveals that there was no significant difference in all measurements before and after receiving the booklet-based training (control group). This result concludes that booklets have no impact on patients’ awareness and behavioral determinants.

### Qualitative Results

#### Overview

We conducted a qualitative thematic analysis to extract key themes from the qualitative data. Braun and Clarke [20] defined thematic analysis as a process of identifying meaningful patterns from qualitative data. Each pattern relates to a theme that is built using the process of coding interview transcripts [20]. Thematic analysis is used in information systems research for different purposes. Information systems researchers adapt thematic analysis to understand interesting phenomena related to information systems [21]. Additionally, thematic analysis is used in the design science paradigm to evaluate the effectiveness of information technology artifacts [22,23].

**Table 1:** Pre-exposure analysis comparing patients’ perceptions between the intervention group and control group.

Measure	Intervention group (n=11), mean score	Control group (n=10), mean score	Mean difference	Levene test		*t* test	
				F_2,19_	*P* value	*t*	*P* value (two-tailed)
Awareness level	3.28	3.33	0.05	5.86	<.01	0.2	.84
Self-efficacy	3.11	4	0.89	3.6	.07	1.57	.13
Perceived severity	3.03	2.52	0.49	2.72	.11	1.82	.08
Behavioral intention	2.91	3.1	0.19	1.11	.30	0.39	.70

**Table 2:** Pre-post comparisons within groups.

Measure	Control (n=10), mean score				Intervention (n=11), mean score			
	Before	After	Difference	*P* value	Before	After	Difference	*P* value
								
Awareness level	3.33	3.31	0.02	.95	3.28	4.56	1.28	.001^a^
Self-efficacy	4	3.66	0.34	.34	3.11	5.56	2.45	.014^a^
Perceived severity	2.52	2.95	0.43	.43	3.03	3.28	0.25	.60
Behavioral intention	3.1	3.6	0.5	.50	2.91	4.55	1.64	.009^a^

^a^These values are significant.

**Table 3:** Comparison between groups on postsurvey measurements.

Measure	Intervention group (n=11), mean score	Control group (n=10), mean score	Mean difference	Levene test		*t* test	
				F_2,19_	*P* value	*t*	*P* value (two-tailed)
Awareness level	4.56	3.31	1.25	3.77	.07	4.79	.001^a^
Self-efficacy	5.56	3.66	1.9	1.62	.22	2.78	.01^a^
Perceived severity	3.28	2.95	0.33	1.23	.28	0.86	.40
Behavioral intention	4.55	3.6	0.95	4.32	.051	2.05	.054

^a^These values are significant.

Four patients participated in our qualitative study. We started the first semistructured interview with Participant 13 and analyzed the transcript of this interview. The finding from the analysis led to the subsequent interview with Participant 3 who revealed more insights for the app’s usability. Subsequently, we conducted two more semistructured interviews with Participants 6 and 14. We stopped conducting more interviews when the saturation point was reached. This saturation point indicated that no more insights were being generated and all emerging concepts were fully explored [24]. The process of analysis started by coding the interview transcripts using a computer-assisted qualitative data analysis system, NVivo (QSR International, Melbourne, Australia). In this study, the coding started by capturing information related to a patient’s empowerment and user experience. We then related and categorized codes that emerged into subthemes to obtain a comprehensive view of the information. Finally, we related subthemes to each other and defined a theme for each subtheme category. This process ended by identifying four themes: patient empowerment, quality of life, user experience, and perceived seriousness.

#### Patient Empowerment

Patient empowerment refers to the process of seeking knowledge about patients’ health and motivating them to take responsibility for their own health [25]. Patient empowerment can be established by increasing patients’ attention to their COPD symptoms. One participant noted that his attention to COPD symptoms increased after using the MyLung app.

It [the app] helped me to pay a little more attention to the symptoms. I am the type that will just ignore them.

P14

The MyLung app not only increases patients’ attention to their COPD symptoms, but also influences caregivers’ attention in order to avoid imminent COPD episodes.

Yeah, it was kind of giving me a warning to kind of pay attention. I had two days of that, and that was the first two days I put him on his antibiotic, and then it stopped giving the warnings after he started to feel a little bit better.

P14

Because of the decline in their daily activity, patients with COPD are socially isolated. The MyLung app encourages caregivers to be more proactive and provide support to their loved ones. The results show that when caregivers interact with the MyLung dashboard, they feel closer to their patients.

I've dealt with a lot of health issues with both...both my husband and my dad...and honestly, this is the scariest one, and so it was nice to go back...When he started not feeling good, I'm looking through everything you had on there.

P14

Patients can be empowered by their COPD physicians through education, counseling, and patient-centered care [26]. Moreover, the COPD physician can empower patients with an early health decision before a COPD exacerbation occurs. This decision can be delivered through a dashboard that presents patients’ symptoms and vitals to the COPD physician. Patients with COPD feel empowered when a COPD physician receives their information daily.

[I]f my doctor was receiving that information, that kind of made me feel a little better. You know, like if he was to see something that wasn't normal, or if something was too high or too low.

P6

Patients with COPD can evaluate their progress in comparison with other patients by tapping on the profile feature in the MyLung app. When a COPD patient is empowered by information about other patients’ progress, he/she will be persuaded to change his/her lifestyle [11].

I liked the profile, overall, because it helped me to see kind of where I was at, at least in comparison to other people.

P3

One participant described how the profile feature aided her in evaluating her progress.

I notice that...my symptoms a lot of times were a little bit worse than others, but...always...[my] mental state, overall, was always better than others.

P14

#### Health-Related Quality of Life

By using the MyLung app, health-related quality of life can be improved for patients with COPD. Health-related quality of life is an outcome that can be determined by patient’s behavior regarding self-management activities. According to Bourbeau [27], behavioral change is considered a major factor for improving health outcomes in patients with COPD.

One participant explained how she changed her behavior to avoid outdoor air pollution.

I try to stay inside. Well, I'm not outside a whole lot anyway, but yeah, if it’s in red I just try to stay inside more than I normally would.

P6

Another patient with COPD changed her health lifestyle after using the MyLung app. She modified her dietary habits and performed more breathing exercises.

Things you didn't know before, you learn, and you can apply them...eating healthier, number one. [And], trying to exercise more.

P3

Health-related quality of life can also be determined by the outcome of the patient’s symptoms. A patient noticed an improvement in her breathing capacity, which was enabled by avoiding risk factors and engaging in breathing exercises provided by the MyLung app.

It [the app] helps me with my breathing...

P6

Similarly, another patient noticed that her COPD symptoms decreased after taking the medication. The notification messages sent to her indicated that her symptom state was higher than normal.

The only thing that has changed is the cough. That decreased a lot once I got the medication. That’s why I changed to that one.

P13

#### User Experience

User experience occurs when a patient interacts with the app. User experience is defined as the consequence of user expectation of system services within which the interaction occurs, including pleasure and enjoyment [28,29].

As noted above, most patients who uses the app are elderly. Thus, an intuitive design can help patients access educational information with the least effort and complete entering their COPD symptoms quickly. Therefore, we ensured that the user experience involved tapping through as few screens as possible.

[T]he education part was the least complicated because everything was just right there.

P14

Although Participant 14 faced some issues in connecting the pulse oximeter device to the app, his overall experience with the app’s usability was positive.

I think overall, it was easy to use. The only issue [I] ever had was sometimes connecting the device on the finger to the thing. It took a while. Sometimes going in and out, to connect. But other than that, it was very easy to use.

P14

The intuitive design led to patient satisfaction with the app content, practicality, and educational module that comprised of texts and videos.

...it’s good educational-wise for people with COPD, so you learn things that you didn't know before. I just thought it was a good...I think it’s a good app.

P3

They're [app features] very helpful and I was very satisfied.

P6

Educational information is delivered to the patients with COPD in many ways to ensure they have reusable content. The app content includes texts, images, and videos and has been organized in the way patients want or need to consume information. One participant explained how she benefited from educational tips by applying them to managing her conditions.

[In]the educational parts, you can go back and watch the educational part and it'll give you tips as to how to manage your breathing, what to do, you know, for COPD exacerbation if you're not knowledgeable, and use those tips and kind of monitor, and use the tips, and they're helpful like that.

P3

Moreover, patients’ experience with the app’s usability can be evaluated by having effective notification messages that warn patients before a COPD exacerbation occurs. These messages can guide patients and their caregivers to take action.

...it [the notification message] helps me monitor my oxygen, and it just seems to be very helpful.

P3

Well, I definitely like the warning because at least I know to keep a closer eye...

P14

Although most patients with COPD were satisfied with the app, two patients encountered some challenges using the pulse oximeter device. These challenges related to both Bluetooth connectivity and reading precision. Occasionally, Participant 14 faced some issues connecting the pulse oximeter device to the app.

The only issue [I] ever had was sometimes connecting the device on the finger to the thing. It took a while. Sometimes going in and out, to connect.

P14

Female patients who were wearing nail polish might have received inaccurate readings from the pulse device, as nail polish can be problematic for obtaining a reliable reading, particularly for patients with COPD who are wearing black, green, or blue nail polish [30]. Over the course of time, one patient using MyLung noticed that her SpO_2_ values were not accurately reflected when she was wearing nail polish. She thought the pulse oximeter device did not work properly.

Well sometimes it doesn't work if you have fingernail polish on...Not annoying, you just have to maneuver your finger.

P3

#### Perceived Seriousness

Patients perceive the seriousness of COPD when they believe that it is life threatening. The consequences of the disease can have a bearing on the quality of life and economic impacts, among other aspects. Perceived severity is a key construct of the HBM. This construct was used in the quantitative study to measure the effectiveness of the MyLung app, as it explains how patients with COPD achieve higher levels of understanding of the seriousness of risks after using the MyLung app. Although the quantitative study showed that using the MyLung had no effect on perceived severity, qualitative interviews were able to gather insight into patients’ perception of disease seriousness.

Patients can respond to the seriousness of the disease by avoiding risk factors that threaten their lives. Participant 6 explained how the MyLung app increased her attention toward outdoor pollution.

Well, I mean...like, if the air is bad just to stay out of it.

P6

Patients also perceived the seriousness of the disease from economic impacts. Patients were afraid of losing their jobs. Patients also began to worry about the increasing cost of health insurance coverage.

I got the intentions to stop, for a couple of reasons. My health and also my insurance. If I stop smoking, then my insurance will go down.

P13

## Discussion

This research study designs and evaluates an assistive technology for patients with COPD to increase awareness levels and engage patients in self-care management activities. The assistive technology aims to inform patients about the risk factors of COPD and to improve access to relevant information. Integrating the findings from quantitative and qualitative analyses led to inferences that described the impact of the design in a comprehensive way. These inferences are referred to as “meta-inferences” [31,32]. According to Venkatesh et al [31], meta-inferences are defined as “the theoretical statements, narratives, or a study inferred from an integration of findings from quantitative and qualitative strands of mixed methods research.” In this study, the inferences that emerged are generalized statements that can be adapted by other researchers interested in assistive technology in respiratory diseases. For enhancement of meta-inferences, we developed the quantitative inferences first and the qualitative inferences second. The results show a great deal of convergence between the quantitative and qualitative inferences, but also reveal some inconsistent findings. Overall, themes that emerged from the qualitative analysis were found to be compatible with the quantitative findings. We found that the design of the MyLung app empowered patients with tools that led to improving their quality of life and increased their intentions in health behavioral tasks. We found that the MyLung app is useable, and patients were satisfied with the app experience. Although the perceived severity of COPD was not significant in the quantitative study, the qualitative analysis revealed a contrasting result: Patients with COPD perceived the severity of the disease when they believed that the consequences of COPD would threaten their life. In addition, the qualitative results provided evidence to show that patients are concerned about the financial burden caused by disease treatment. Considering the integrated findings or inferences from both analyses, we arrived at the following meta-inferences: (1) The design of assistive technology for patients with COPD will empower patients with a mechanism that improves patients’ quality of life and increase patients’ intention toward health behavioral tasks. (2) The design of assistive technology for patients with COPD is technically usable.

Our findings and meta-inferences have implications for assistive technology design, research on chronic condition management, and health care. The results of this study can inform research on health information design. Meta-inferences act as propositions that give researchers a useful starting point and direction for future research to understand and investigate phenomena related to research in chronic disease management information systems. Researchers should investigate the role of design in integrative mobile-based technology for patient empowerment and health outcomes.

## Abbreviations

BCSS: behavioral change-support system
COPD: chronic obstructive pulmonary disease
HBM: health belief model
mHealth: mobile health
SpO_2_: peripheral capillary oxygen saturation
UCOPD: understanding chronic obstructive pulmonary disease
